# The Importance of Early Suspicion for Cold Autoimmune Hemolytic Anemia

**DOI:** 10.7759/cureus.49160

**Published:** 2023-11-21

**Authors:** Naisarg B Vanani, Eric Bejarano, Andrea Bequest, Douglas Levine

**Affiliations:** 1 Internal Medicine, Medical College of Wisconsin, Milwaukee, USA

**Keywords:** mortality and morbidity reduction, critical care and hospital medicine, severe anemia, autoimmune hemolytic anemia (aiha), cold agglutinin disease

## Abstract

Cold autoimmune hemolytic anemia (cAIHA) is a form of autoimmune hemolytic anemia (AIHA) that most often involves agglutinin antibodies that specifically react to cold temperatures. This process most commonly involves an immunoglobulin M (IgM)-mediated agglutination of erythrocytes and can result in complement-mediated hemolysis, which can range greatly in severity from case to case. Here, we present a case of cAIHA in a 64-year-old male who presented with rapidly progressive and severe hemolytic anemia, which resulted in irreversible decompensation. This case highlights the importance of maintaining a high index of suspicion for cAIHA in patients older adult patients with a previous history of autoimmune hematologic diseases presenting in a rapidly progressive hemolytic state, which can allow for prompt diagnosis, treatment, and mitigation of adverse outcomes.

## Introduction

Autoimmune hemolytic anemias (AIHAs) are generally categorized into three subtypes: cold, mixed, and warm hemolytic anemias. Cold agglutinin disease (CAD) is a rare form of cold AIHA (cAIHA) that has an estimated incidence of 0.8-3/100,000 per year. Northern regions, such as Norway, have slightly higher rates [1,2]. CAD is further subcategorized as CAD, paroxysmal cold hemoglobinuria, or secondary cold AIHAs usually secondary to malignancies [1]. In cases of suspected cold autoimmune hemolysis, the potential for severe hemolysis with associated anemia and end-organ damage makes quick diagnosis and treatment necessary to prevent long-term complications and minimize mortality [3]. Here, we present a case of cold agglutinin hemolytic anemia that highlights the indispensability of prompt diagnostic workup and treatment with ICU-based care in cases of suspected cAIHA complicated by severe hemolysis, end-organ damage, and ultimately, mortality.

## Case presentation

Here, we present a 64-year-old male with a medical history of gastroesophageal reflux disease (GERD) and immune thrombocytopenic purpura (ITP). He had undergone splenectomy previously and presented with complaints of dark urine, along with bilateral hand paresthesias and *kaleidoscope-like* visual disturbances. The patient reported an episode of dark urine earlier in the day preceding his presentation, along with new-onset bilateral tingling in his hands. He also reported an episode of Melena the day before his presentation. The patient denied any loss of consciousness, aphasia, or weakness. At the time of his presentation, the patient described persistently *dark urine*. He had been taking a daily multivitamin with iron for the past 25 years, along with omeprazole (20 mg/day) for acid reflux, and rosuvastatin (10 mg/day) for hyperlipidemia.

The review of systems (ROS) on admission revealed paresthesias in the bilateral upper extremities (R > L), with the rest of the ROS and physical exam being unremarkable. The initial differential diagnosis was broad, but diagnoses such as nephrotic syndrome, hemolysis, rhabdomyolysis, hypercalciuria, cerebral vascular accident, and transient ischemic attack were considered. A computed tomography (CT) with angiography was negative for any acute intracranial findings and found no evidence of large vessel occlusion, significant stenosis, dissection, or aneurysm. Additionally, CT of the abdomen and pelvis with contrast was negative for any acute processes. Pertinent laboratory data are displayed in Table 1. 

**Table 1:** Pertinent labs throughout the hospital course. IgM, immunoglobulin M; IgG, immunoglobulin G; IgA, immunoglobulin A; ---, lab not collected

Pertinent lab tests (Units)	Hospital day 1	Hospital day 2	Reference range
Hemoglobin (g/dL)	8.9	6.4	13.7-17.5
Haptoglobin (mg/dL)	<10	<10	30-200
Total bilirubin (mg/dL)	2.8	2.9	0.2-1.2
Lactate dehydrogenase (U/L)	1,597	3,576	135-225
Leukocyte count (1,000 microL^-1^)	13.8	14.2	3.9-11.2
Reticulocyte (%)	6.4	9.1	0.9-2.7
Platelet count (1,000 microL^-1^)	349	289	165-366
Creatine kinase (U/L)	363	---	<190
Serum IgM (mg/dL)	39	---	40-230
Serum IgG (mg/dL)	1164	---	700-1600
Serum IgA (mg/dL)	387	---	70-400
Alkaline phosphatase (U/L)	63	50	40-129
Aspartate aminotransferase (U/L)	---	404	13-44
Creatinine (mg/dL)	1.1	1.36	0.70-1.30

**Figure 1:**
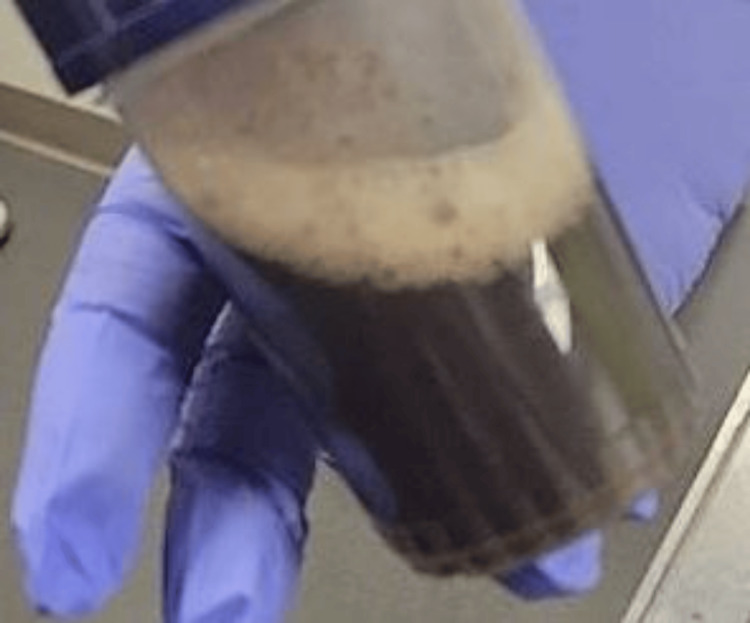
Dark urine collected on admission.

Laboratory data were significant for a decreased hemoglobin, which had dropped from a previous baseline of 13-14 mg/dL. Additional laboratory studies were consistent with a hemolytic mechanism, including elevated total bilirubin, undetectable haptoglobin, elevated lactate dehydrogenase (LDH), and an appropriate reactive reticulocytosis. There was also mild leukocytosis at this time with a normal platelet count. Urinalysis revealed nearly black urine with the presence of blood, hemoglobin, and scant bacteria, without WBC casts (Figure 1). Thus, the patient's presentation at this point was most consistent with a hemolytic anemia of unclear etiology, and he was admitted for further evaluation and treatment.

On hospital day 1, the patient was found to be jaundiced, icteric, febrile at 100.9 °F (38.3 °C), slightly tachycardia at 90-110 beats/min, and hypertensive at 195/66 mmHg. Hematology was consulted for further evaluation of the patient’s condition. Further testing initially revealed a negative direct agglutination (DAG) test at 37-38 °C. However, upon cooling the sample to 3-4 °C, antibodies reacting to these cold temperatures and causing agglutination were observed. Additional lab testing resulted in a decreased level of serum IgM, with a negative paroxysmal nocturnal hemoglobinuria blood screening. Due to concern for potential acute kidney injury (AKI), intravenous (IV) fluids were administered, and 1 unit of blood was transfused. However, 30 minutes following this transfusion, the patient developed acute respiratory distress, initially experiencing oxygen desaturation to 70% SpO_2_, with subsequent improvement upon receiving supplemental oxygen.

On hospital day 2, laboratory results showed a further drop in hemoglobin. Due to the patient’s previous reaction to blood products and suspicion of cold agglutinin etiology, transfusing the patient with warmed and crossmatched blood products was considered to avoid further exacerbating cold agglutination with standard unwarmed products. However, the process for obtaining warmed blood products, along with the time required for warming, was found to be too long, even considering uncrossmatched products. For this reason, the patient initially received standard unwarmed blood transfusions. Throughout hospital day 2, the patient had increasing oxygen requirements and remained persistently tachycardic. Several attempts were made to collect laboratory samples, but they were unsuccessful because the samples became hemolyzed. Chest radiographs and CT scans showed no evidence of pulmonary infiltrates, thus lowering suspicion of transfusion-related acute lung injury (TRALI), transfusion-associated circulatory overload (TACO), or true transfusion reaction. For this reason, the patient's increasing oxygen demand was deemed to most likely be secondary to the active aggressive hemolytic process and resulting tissue hypoperfusion.

Due to suspicion that his anemia was progressing further, the patient was transferred to the medical intensive care unit for potential plasmapheresis, monitoring, and continued respiratory support. Shortly after his transfer to the ICU, the patient became bradypneic and unresponsive and was found to be in pulseless electrical activity (PEA) arrest. Due to the patient’s rapid deterioration, the warmed blood products were not prepared in time, and given the setting of autoimmune hemolysis, uncrossed packed red blood cell (pRBC) infusions were deemed to cause further harm and were deferred. During the resuscitation, calcium gluconate was administered to the patient due to concern for hyperkalemia in the setting of hemolysis. Return of spontaneous circulation was eventually achieved after 20 minutes of resuscitation efforts. However, approximately 1 minute later, the patient once again deteriorated into PEA arrest and eventually asystole. After 30 minutes of resuscitation, the decision was made by the family to terminate resuscitation efforts. 

This course of decompensation was deemed to be secondary to end-organ damage in the setting of hypoperfusion due to severe ischemia from low-circulating RBCs. On hospital day 2, the patient was also found to have newly elevated liver enzymes, with aspartate aminotransferase (AST) elevated from a previously normal baseline. Further laboratory values surrounding the patient’s decompensation are displayed in Table 2. Several hours before the patient’s PEA arrest, the patient’s lactic acid level was found to be markedly elevated, further demonstrating the extent of tissue hypoxia and impending end-organ failure. This was further elucidated by the patient’s sudden spike in N-terminal prohormone of brain natriuretic peptide (NT-proBNP) in the setting of subsequent heart failure. At the time of PEA arrest, the patient’s arterial blood gas (ABG) revealed a severely hypercapnic, hypoxemic, and acidotic picture. Before this decompensation, a euvolemic exam, clear chest radiograph, and a reassuring ABG with adequate oxygenation indicated that hypoxia from poor ventilation or diffusion was a less likely cause of the patient’s end-organ failure but was likely driven by poor oxygen delivery to tissues secondary to severe anemia.

**Table 2:** Pertinent labs surrounding decompensation. NT-proBNP, N-terminal prohormone of brain natriuretic peptide; PEA, pulseless electrical activity; ---, lab not collected

Lab tests (Units)	Before PEA arrest	During PEA arrest	Reference range
Lactic acid (mmol/L)	10.5	---	0.5-2.0
NT-proBNP (pg/mL)	596	2,962	<125
Arterial pH	7.45	6.88	7.35-7.45
Arterial pCO_2 _(mmHg)	25	71	35-45
Arterial pO_2_ (mmHg)	314	<20	80-104
Arterial HCO_3_ (mmol/L)	18	15	21-31

## Discussion

CAD is a rare condition that typically manifests in older adults. A retrospective study involving 89 patients reported a median age of symptom onset at 65 and a median age of diagnosis at 72 [4]. The pathophysiology of CAD involves a reaction between erythrocyte surface antigens and antibodies in the setting of lower temperatures such as those seen in the extremities. The presentation of CAD can range from mild cases, which are largely asymptomatic to severe anemias, with rare cases resulting in organ failure [5,6]. Furthermore, the most common presentation of CAD can result in cold-induced findings, especially in areas of the body that are more prone to cold exposure such as the extremities often causing livedo reticularis, Raynaud’s phenomenon, and cyanosis, among others [6]. 

While fatal cases of hemolytic anemias are most commonly reported to be caused by drug-induced factors, and conditions such as angina and heart failure are attributed to warm antibody hemolytic anemia, fatal cases of hemolytic anemia specifically involving CAD have not been as extensively reported in the literature [7]. CAD has been shown to increase the risk of thrombosis, with a relative risk of 3.1 in a retrospective study of 608 CAD patients over 10 years, more commonly causing venous events over arterial thrombosis and myocardial infarctions [8]. Additionally, while cases of renal failure have been reported to be caused by autoimmune hemolytic anemias, our case involves cardiovascular failure, which has been poorly characterized by CAD [9]. Thus, to the best of our knowledge, our case is the first to report such a rapidly progressive, fatal hemolytic anemia resulting in tissue hypoxia secondary to severe anemia, lactic acidosis, and eventual end-organ heart failure caused by CAD. 

In a multinational study of 232 patients with CAD, 12% of patients had compensated anemia, 24% were classified to have mild anemia with hemoglobin levels of 10 g/dL to lower limit of normal, 37% had moderate anemia (8-10 g/dL), and 27% of patients having severe anemia with hemoglobin levels less than 8 g/dL [10]. Additionally, in this study, patients in the severe cold agglutinin anemia category were found to have an elevated LDH average of 534 U/L, with decreased IgM titers with an average of 5.5 g/L. This study highlights the novelty of our patient’s case as he presented with rapidly progressive hemolysis and a hemoglobin of 6.4 g/dL on hospital day 2. Compared to the averages found in this study, our patient had an LDH of 2,162 U/L on initial presentation, with an undetectable haptoglobin level, and an IgM titer of 0.39 g/L, which represents a severe case of cAIHA, even concerning the severe category. 

In the case of CAD, IgM antibodies are involved in cold agglutination in most cases, whereas IgG antibodies have been mostly implicated in warm agglutination with only rare cases of IgG-mediated cold agglutination [11,12]. In most cases of CAD, these IgM autoantibodies bind to erythrocyte antigens, forming antibody-antigen complexes that activate the classical complement pathway via C1-esterase activation of C2 and C4, leading to complement-mediated hemolysis [11,13]. Additionally, while warming can cause the dissociation of these IgM complexes, it does not cause the dissociation of the antigens involved in complement activation. The retention of these erythrocyte antigens involved in complement activation causes a positive direct Coombs test, which is often suggestive of CAD [14,15]. In our case, due to the severity of the hemolysis caused by CAD, the direct Coombs test was falsely negative. However, upon cooling the sample to 3-4 °C, agglutination was observed. This finding, along with the low IgM titer, suggested IgM coating RBCs was causing the cold agglutinin hemolytic anemia and corroborated our suspicion of CAD. It is important to note that infection with Mycoplasma pneumoniae, a common cause of atypical pneumonia, can also be a cause of IgM-mediated CAD [14]. However, the absence of imaging consistent with mycoplasma pneumonia, and negative mycoplasma lab results point to a likely etiology of primary CAD. 

With the potential of rapid hemolysis and concern for end-organ damage, especially in colder climates, the timely diagnosis and treatment of cAIHA is crucial to patient survival and salvaging long-term organ function in severe cases. Thus, while cAIHA is a relatively rare phenomenon, it is vital to maintain a high index of clinical suspicion for cAIHA, especially in cases where the patient has a past medical history of autoimmune disorders and hematologic conditions such as immune thrombocytopenic purpura (ITP) as seen in our patient [15]. In a case of cAIHA presenting in the setting of atypical pneumonia, the patient initially appeared with a much lower initial hemoglobin level of 2.9 g/dL, compared to our case at 8.9 g/dL [16]. In this case, the patient was initially treated with regular pRBCs, which gave an initial improvement of hemoglobin to 6.2 g/dL, which dropped back to 4.2 g/dL by the following day. This is similar to our case in which attempts to correct hemoglobin via pRBC infusions were reversed by the active cAIHA process. This patient was then given six units of warmed pRBCs, which were able to correct and maintain the patient’s hemoglobin level above 7 g/dL. This case demonstrates the potential value of warmed RBC products for cases of CAD where standard products fail to correct the patient’s hemolysis, which could have been of value for our case if the warmed products were promptly available. Other cases of CAD presenting as cAIHA demonstrated a similar presentation as our case, with fatigue, jaundice, dark urine consistent with hemoglobinuria, and general malaise. However, unlike our case, both cases had a stable hospital course and were ultimately resolved with high-dose steroids during inpatient care, leading to discharge on a course of oral steroids [17,18]. As can be seen in our case, the delay in diagnosis and lab results due to severe hemolysis allowed for further rapid progression of our patient’s condition. In this instance, we posit that prompt initiation of ICU-level care and targeted interventions could have minimized the risk, morbidity, and mortality. 

With this in mind, it is important to discuss the various treatment options that can be considered for cAIHA with significant anemia. Common initial treatments for patients with few preexisting comorbidities include a dual therapy with rituximab-bendamustine, where complement inhibitors may be added to the treatment regimen for lack of response. On the other hand, patients with comorbidities, as in our case, are often started initially on rituximab monotherapy with a complement inhibitor or bortezomib for lack of response to initial therapy [6]. There have also been recent trials showing sutimlimab as a long-term treatment for CAD. Sutimlimab is a humanized monoclonal antibody targeting the complement pathway by inhibiting the C1 complex serine protease. In a 26-week trial involving 24 patients with CAD, the therapy resulted in a 2.6 g/dL increase in mean hemoglobin among all subjects by the end of the trial, demonstrating efficacy as a long-term treatment for anemias secondary to CAD [19]. While these medications offer long-term maintenance therapy, flare-ups can occur, for which short-term therapy such as plasmapheresis along with eculizumab are generally preferred during inpatient stabilization and treatment [20]. Thus, short- and long-term treatment options are available for CAD and can be tailored to the patient’s specific comorbidities. Given this, the most crucial factors in effective treatment include maintaining a higher degree of suspicion to allow for early diagnosis, prompt empiric treatment, and early ICU management for patients presenting with fulminant hemolysis in cases like ours. Given the rare nature of the disease, future research targeting the antigenic markers that trigger the IgM-mediated agglutination seen in cAIHA will enable focused treatment of CAD with fewer off-target effects, especially in patients with multiple comorbidities.

## Conclusions

Autoimmune hemolytic anemias, including cAIHA, can have a variable presentation, and, if not addressed quickly, can lead to rapid clinical deterioration. Thus, it is important to maintain a high index of clinical suspicion for cAIHA, especially in middle-aged to older adults with a prior history of autoimmune hematological conditions. If discovered earlier, patients could be initiated on life-saving treatment to prevent starting a cascade of irreversible clinical deterioration. Further research is needed to investigate protocols that could expedite the readiness of warmed blood products as this could be a life-saving measure where hours and minutes could mean the difference between life and death.

## References

[1] Gabbard AP, Booth GS (2020). Cold agglutinin disease. Clin Hematol Int.

[2] Berentsen S, Ulvestad E, Langholm R (2006). Primary chronic cold agglutinin disease: a population based clinical study of 86 patients. Haematologica.

[3] Berentsen S (2016). Cold agglutinin disease. Hematology Am Soc Hematol Educ Program.

[4] Swiecicki PL, Hegerova LT, Gertz MA (2013). Cold agglutinin disease. Blood.

[5] Poldre P, Pruzanski W, Chiu HM, Dotten DA (1985). Fulminant gangrene in transient cold agglutinemia associated with Escherichia coli infection. Can Med Assoc J.

[6] Berentsen S (2021). How I treat cold agglutinin disease. Blood.

[7] Packman CH (2015). The clinical pictures of autoimmune hemolytic anemia. Transfus Med Hemother.

[8] Broome CM, Cunningham JM, Mullins M, Jiang X, Bylsma LC, Fryzek JP, Rosenthal A (2020). Increased risk of thrombotic events in cold agglutinin disease: a 10-year retrospective analysis. Res Pract Thromb Haemost.

[9] Habibi B, Basty R, Chodez S, Prunat A (1985). Thiopental-related immune hemolytic anemia and renal failure. Specific involvement of red-cell antigen I. N Engl J Med.

[10] Berentsen S, Barcellini W, D'Sa S (2020). Cold agglutinin disease revisited: a multinational, observational study of 232 patients. Blood.

[11] Baines AC, Brodsky RA (2017). Complementopathies. Blood Rev.

[12] Silberstein LE, Berkman EM, Schreiber AD (1987). Cold hemagglutinin disease associated with IgG cold-reactive antibody. Ann Intern Med.

[13] Zilow G, Kirschfink M, Roelcke D (1994). Red cell destruction in cold agglutinin disease. Infus Ther Transfus Med.

[14] Hu J, Ye Y, Chen X, Xiong L, Xie W, Liu P (2022). Insight into the pathogenic mechanism of Mycoplasma pneumoniae. Curr Microbiol.

[15] Barcellini W, Fattizzo B (2023). Strategies to overcome the diagnostic challenges of autoimmune hemolytic anemias. Expert Rev Hematol.

[16] Jalal Eldin A, Thomas R, Gibson G (2023). Hemolytic anemia in the setting of atypical pneumonia: a case of cold agglutinin disease. Cureus.

[17] Guevara NA, Perez E, Sanchez J, Rosado F, Sequeira Gross HG, Fulger I (2023). A case report of cold agglutinin disease, severe B12 deficiency, and pernicious anemia: a deadly coincidence. Cureus.

[18] Brazel D, Eid T, Harding C (2021). Warm and cold autoimmune hemolytic anemia in the setting of COVID-19 disease. Cureus.

[19] Röth A, Barcellini W, D'Sa S (2021). Sutimlimab in cold agglutinin disease. N Engl J Med.

[20] Murakhovskaya I (2020). Rituximab use in warm and cold autoimmune hemolytic anemia. J Clin Med.

